# Preoperative Prediction of Perineural Invasion Status of Rectal Cancer Based on Radiomics Nomogram of Multiparametric Magnetic Resonance Imaging

**DOI:** 10.3389/fonc.2022.828904

**Published:** 2022-04-11

**Authors:** Yang Zhang, Jiaxuan Peng, Jing Liu, Yanqing Ma, Zhenyu Shu

**Affiliations:** ^1^ Cancer Center, Department of Radiology, Zhejiang Provincial People’s Hospital, Affiliated People’s Hospital, Hangzhou Medical College, Hangzhou, China; ^2^ Medical College, Jinzhou Medical University, Jinzhou, China

**Keywords:** rectal cancer, perineural invasion, multiparametric magnetic resonance imaging, radiomics, nomogram

## Abstract

**Objectives:**

To compare the predictive performance of different radiomics signatures from multiparametric magnetic resonance imaging (mpMRI), including four sequences when used individually or combined, and to establish and validate an optimal nomogram for predicting perineural invasion (PNI) in rectal cancer (RC) patients.

**Methods:**

Our retrospective study included 279 RC patients without preoperative antitumor therapy (194 in the training dataset and 85 in the test dataset) who underwent preoperative mpMRI scan between January 2017 and January 2021. Among them, 72 cases were PNI-positive. Then, clinical and radiological variables were collected, including carcinoembryonic antigen (CEA), radiological tumour stage (T_1-4_), lymph node stage (N_0-2_) and so on. Quantitative radiomics features were extracted and selected from oblique axial T_2_-weighted imaging (T_2_WI), T_1_-weighted imaging (T_1_WI), apparent diffusion coefficient (ADC), and enhanced T_1_WI (T_1_CE) sequences. The clinical model was constructed by integrating the final selected clinical and radiological variables. The radiomics signatures included four single-sequence signatures and one fusion signature were built using the respective remaining optimized features. And the nomogram was constructed based on the independent predictors by using multivariable logistic regression. The area under curve (AUC), DeLong test, calibration curve, and decision curve analysis (DCA) were used to evaluate the performance.

**Results:**

Ultimately, 20 radiomics features were retained from the four sequences—T_1_WI (n = 4), T_2_WI (n = 5), ADC (n = 5), and T_1_CE (n = 6)—to construct four single-sequence radiomics signatures and one fusion radiomics signature. The fusion radiomics signature performed better than four single-sequence radiomics signatures and clinical model (AUCs of 0.835 and 0.773 vs. 0.680-0.737 and 0.666-0.709 in the training and test datasets, respectively). The nomogram constructed by incorporating CEA, tumour stage and rad-score performed best, with AUCs of 0.869 and 0.864 in the training and test datasets, respectively. Delong test showed that the nomogram was significantly different from the clinical model and four single-sequence radiomics signatures (*P* < 0.05). Moreover, calibration curves demonstrated good agreement, and DCA highlighted benefits of the nomogram.

**Conclusions:**

The comprehensive nomogram can preoperatively and noninvasively predict PNI status, provide a convenient and practical tool for treatment strategy, and help optimize individualized clinical decision-making in RC patients.

## Introduction

Rectal cancer (RC) is one of the most common cancers and the leading cause of death, and its incidence is on the rise worldwide (1, 2). RC patients are usually in the middle and late stages when diagnosed, for which the standard treatment recommended by the National Comprehensive Cancer Network guidelines is preoperative neoadjuvant chemoradiotherapy (nCRT) combined with total mesorectal resection (3). MRI has an important role associated with biopsy data for preoperative planning and the choice of undergoing chemoradiotherapy. Perineural invasion (PNI) has been recognized as an independent prognostic factor in RC patients since the 7th edition of the Tumour Node Metastasis (TNM) classification system (4).

PNI is defined as tumour cells growing around, within, or through any of the three nerve layers and should surround more than 33% of the nerve circumference (5). Studies found that PNI was associated with a significantly poorer prognosis, which may be due to the presence of tumour cells located in the nerve bundles that cannot be sufficiently removed by radical surgery and that lead to disease recurrence (6–8). For this reason, PNI was introduced as an accessory factor and has been suggested as a prognostic factor to help select patients who may benefit from nCRT (9, 10). Therefore, accurate preoperative assessment of PNI status is helpful for clinical management and prognostic prediction.

However, conventional preoperative biopsy usually only detects the mucosal and submucosal layers, while peripheral nerves generally exist outside the mucosal muscle layer and partly outside the intestinal wall, so biopsy cannot accurately detect PNI status (11). Currently, PNI assessment is mainly dependent on postoperative pathological examination, but its efficiency and timeliness limit its application (12, 13). Multiparametric magnetic resonance imaging (mpMRI), as an important part of preoperative examination in clinical practice, has been used as the main noninvasive method for preoperative evaluation of RC patients, but unfortunately, it cannot show tiny peripheral nerves (14). Therefore, it is necessary to find a reliable way to provide PNI-related information before clinicians make treatment decisions.

Radiomics uses big data mining techniques to analyse the correlation between radiological features and pathological data. Therefore, it is a powerful tool to provide oncology decision support (15–17). Several recent studies have shown that radiomics is a superior tool for predicting the occurrence of PNI in colorectal cancer (18, 19). It is worth noting that this type of research only uses CT images with radiation damage as the analysis object. In addition, Yang et al. (20) found that T_2_WI-based radiomic nomogram could be helpful in the prediction of preoperative PNI in RC patients. However, noninvasive mpMRI includes different sequences, indicating its greater potential to provide more useful information (21). The high-latitude analysis of mpMRI was used to extract relevant radiomics features and integrate clinical data to further establish the combined model, which can supplement the deficiency of traditional visual evaluation and help clinicians predict the PNI status in RC patients. In addition, predictive and prognostic models of radiomics are also important in clinical practice, and highly accurate and reliable models are needed to improve the decision-making process.

Therefore, we aimed to systematically evaluate and compare the predictive performance for PNI in RC patients based on radiomics from mpMRI, including T_2_-weighted imaging (T_2_WI), T_1_-weighted imaging (T_1_WI), apparent diffusion coefficient (ADC), and enhanced T_1_WI (T_1_CE) sequences when used individually or combined, to obtain the optimal radiomics signature and construct nomogram in combination with PNI related clinical data in order to provide a basis for disease management strategies.

## Materials and Methods

### Patients

This retrospective study was approved by our institutional ethics committee, and the requirement for written informed consent was waived.

**Figure 1:**
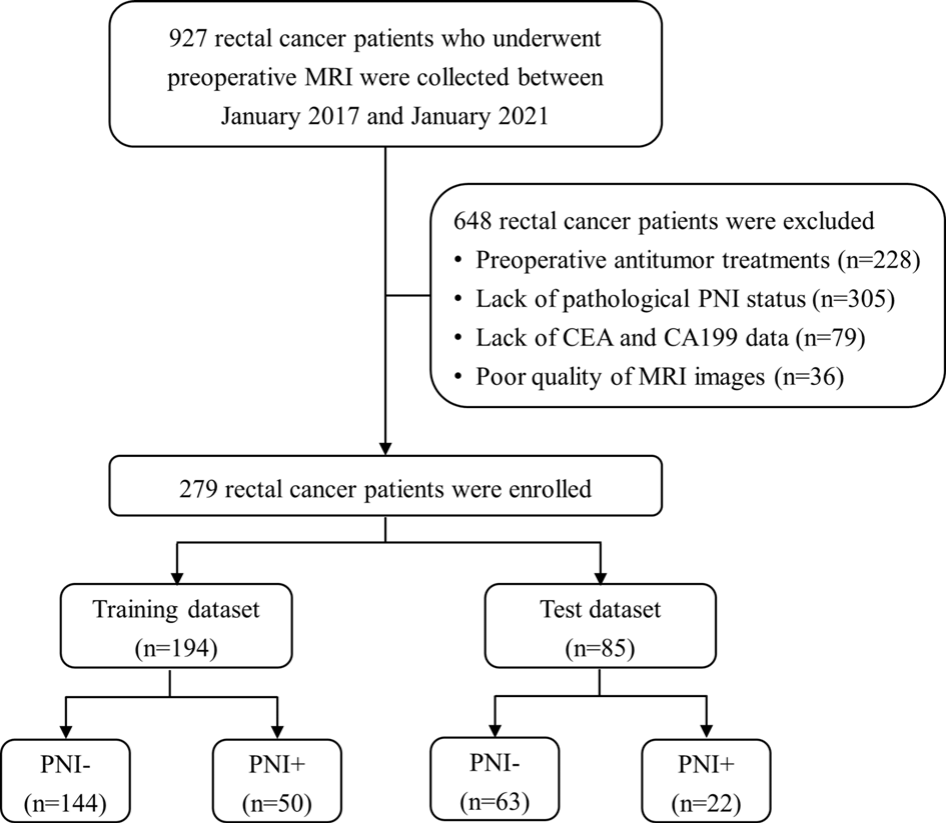
Patient recruitment process. (PNI-, patients without pathologic perineural invasion; PNI+, patients with pathologic perineural invasion; CEA, carcinoembryonic antigen; CA19-9, carbohydrate antigen 19-9).

In this study, 927 RC patients who underwent preoperative MRI were collected between January 2017 and January 2021 from our picture archiving and communication system (PACS). And 648 patients were excluded for the following reasons: (a) preoperative antitumor treatments (*n* = 228), (b) lack of pathological PNI status (*n* = 305), (c) lack of CEA and CA19-9 data (*n* = 79), and (d) poor quality of MRI images (*n* = 36). Finally, 279 patients with histologically confirmed RC were enrolled and divided into training (*n* = 194) and test (*n* = 85) datasets at a ratio of 7:3. The training set was used for model construction, and the test set was used for validation. The patient selection process is shown in **Figure 1**.

The preoperative information on clinical and radiological variables was retrospectively collected from our PACS. Clinical variables included age, gender, carcinoembryonic antigen (CEA), and carbohydrate antigen 19-9 (CA19-9). The radiological variables included MRI-based extramural vascular invasion (mrEMVI) status, circumferential resection margin (CRM) status, distance between tumour and anal margin (DIS), radiological tumour stage (T_1-4_) and lymph node stage (N_0-2_). Data on PNI status were obtained from pathological reports, and the patients were divided into two groups: PNI-positive group (PNI+) and PNI-negative group (PNI-). Detailed information can be found in **Supplementary Data 1**.

### MRI Scan

All patients underwent MRI examinations using a 3.0 T MRI scanner (Skyra; Siemens Healthineers) equipped with an 8-channel phased-array coil in the supine position. The rectal MRI sequences included sagittal localizing T_2_WI, axial T_2__blade_TSE, axial T_1_WI, axial diffusion-weighted imaging (DWI) with b value = 1000 s/mm^2^, ADC, and T_1_CE. The gadolinium-based agent (Magnevist; Bayer Healthcare, Berlin, Germany) was intravenous injected using an MRI compatible power injector at a rate of 2 mL/s and a dose of 0.2 mL/kg of body weight, followed by a 20 mL saline flush with the high-pressure injector in order to obtain T_1_CE sequence. As previously reported (20), the sequences on the axial plane were collected on an oblique-axial plane perpendicular to the long axis of the RC. The detailed parameters for each sequence are illustrated in **Table 1**.

**Table 1:** MRI parameters of each sequence.

Scanner	Sequence	Orientation	TR (ms)	TE (ms)	FOV (mm^2^)	Thickness (mm)	Interslice gap (mm)	Matrix	NEX
SIEMENS 3.0T (Skyra)	T_2_WI	Sagittal	6060	90	180×180	3	0.6	320×224	2
	T_2_WI	Axial	4790	134	200×200	3	0.6	384×451	2
	T_1_WI	Axial	662	9.6	180×180	3	0.6	320×224	1
	DWI	Axial	7330	56.0	200×200	3	0.8	112×100	1
	T_1_CE	Axial	616	9.6	180×180	3	0.6	320×224	1

TR, repetition time; TE, echo time; FOV, field of view.

### Image Preprocessing and Segmentation

Before segmentation, image preprocessing and registration were performed using A.K. software (Analysis Kit, GE Healthcare). Of which, the image registration function was used to adopt the oblique-axial T_2_WI sequence as the template for rigid registration of all sequences to ensure that sequences contained the same resolution, spacing, and origin. Two radiologists (senior radiologist and junior radiologist) with 13 and 8 years of experience in rectal MRI independently used ITK-SNAP software (www.itksnap.org) to perform three-dimensional manual segmentations of the entire tumour. Specifically, the standardized T_2_WI sequence was used to segment the entire rectal tumour slice-by-slice to determine the volume of interest (VOI). Depending on the registration, T_1_WI, ADC, and T_1_CE can share the same VOI obtained from T_2_WI. More information can be found in **Supplementary Data 2**.

### Radiomics Features Extraction and Selection

All VOIs were imported into A.K. software for feature extraction. And 396 radiomic features were extracted for each patient each sequence, including 42 histogram features, 144 gray-level co-occurrence matrix features (GLCM), 11 gray-level size zone matrix features (GLSZM), 180 run-length matrix features (RLM), 9 formfactor features, and 10 haralick features. Detailed information on all radiomics features is described in **Supplementary Data 3**. Then, feature set A (from the senior radiologist) and feature set B (from the junior radiologist) were obtained. For the reproducibility analysis, Spearman’s rank correlation test was used to calculate the intraclass correlation coefficient (ICC) of each feature in sets A and B, and only the features with ICCs greater than 0.80, indicating excellent reproducibility and stability, were included for the dimension reduction step. Analysis of variance and the least absolute shrinkage and selection operator were used to choose the optimized subset of features. Detailed dimension reduction step is described in **Supplementary Data 4**.

### Model Construction and Evaluation

Logistic regression analysis was undertaken to construct one clinical model and five radiomics signatures. The clinical model was constructed by integrating the final selected clinical and radiological variables. The radiomics signatures included four single-sequence signatures based on T_2_WI, T_1_WI, ADC, and T_1_CE, respectively, and one fusion signature based on the four sequences were built using the respective remaining optimized features. Meanwhile, calculating the scores of each patient was used to quantify the identifiability of the radiomics signatures. This reflected the likelihood of PNI and was defined as the radiomics score (rad-score). Considering the potential value of clinical and radiological variables, multivariate logistic regression analysis was used to construct the combined model. Specifically, multivariate logistic regression analysis and a backward stepwise selection method with the stopping rule based on Akaike’s information criterion were conducted to select independent predictors from clinical and radiological variables and an optimized radiomics signature on training data. Finally, the combined model was constructed based on the independent predictors.

The discrimination performance of different models in both the training and test datasets was assessed using the area under the receiver operating characteristic (ROC) curve (AUC) and DeLong test. The calibration curve was used to assess the consistency, and decision curve analysis (DCA) was applied to measure the clinical usefulness of the combined model in predicting PNI. Finally, we developed a visual nomogram to calculate the probability of PNI for each patient based on the combined model. A Sankey plot was used to show the relationship between independent predictors and pathological PNI status.

### Statistical Analysis

Statistical analyses were performed with MedCalc (version 11.2), R (version 3.4.1), and OriginPro (version 9.6.5). Continuous variables were statistically evaluated using a two-sample T test or Mann–Whitney U test according to the distribution of the variables and are presented as the mean ± standard deviation or median (interquartile range). Categorical variables were analysed using the chi-square test and are expressed as numbers (percentages). The statistical significance was set at *P* < 0.05.

## Results

### Patients’ Characteristics

There were no significant differences between the training dataset and test dataset in terms of all parameters (*P* > 0.05). In contrast, we observed significant differences between the PNI+ and PNI− groups in terms of CEA, CRM, and tumour stage in the training dataset (*P* < 0.05), confirmed in the test dataset. In addition, there were significant differences between the two groups in terms of lymph node (*P* = 0.030) in the training dataset and CA19-9 (*P* = 0.025) in the test dataset, as shown in **Table 2**.

**Table 2:** Patients’ characteristics in the training and test datasets.

Variables	Training dataset (n=194)			Test dataset (n=85)			
	PNI- (n=144)	PNI+ (n=50)	*P_intra_*	PNI- (n=63)	PNI+ (n=22)	*P_intra_*	*P_inter_*
Age (years, SD)	66.21 (9.8)	64.16 (9.7)	0.203	63.35 (10.7)	63.59 (7.8)	0.923	0.078
Gender (N, %)							
Male	103 (71.5)	31 (62.0)	0.209	47 (74.6)	18 (81.8)	0.492	0.208
Female	41 (28.5)	19 (38.0)		16 (25.4)	4 (18.2)		
CEA (N, %)							
Abnormal	45 (31.3)	27 (54.0)	0.004^*^	20 (31.7)	13 (59.1)	0.023^*^	0.786
Normal	99 (68.7)	23 (46.0)		43 (68.3)	9 (40.9)		
CA19-9 (N, %)							
Abnormal	11 (7.6)	8 (16.0)	0.151	4 (6.3)	6 (27.3)	0.025^*^	0.620
Normal	133 (92.4)	42 (84.0)		59 (93.7)	16 (72.7)		
DIS (cm, SD)	8.11 (3.3)	7.15 (4.3)	0.151	8.20 (4.0)	7.86 (4.7)	0.740	0.608
CRM status (N, %)							
Positive	29 (20.1)	20 (40.0)	0.005^*^	7 (11.1)	9 (40.9)	0.006^*^	0.242
Negative	115 (79.9)	30 (60.0)		56 (88.9)	13 (59.1)		
mrEMVI status (N, %)							
Positive	28 (19.4)	16 (32.0)	0.068	14 (22.2)	8 (36.4)	0.192	0.562
Negative	116 (80.6)	34 (68.0)		49 (77.8)	14 (63.6)		
Tumor stage (N, %)							
T_1-2_	48 (33.3)	3 (6.0)	0.000^*^	22 (34.9)	2 (9.1)	0.020^*^	0.736
T_3-4_	96 (66.7)	47 (94.0)		41 (65.1)	20 (90.9)		
Lymph node (N, %)							
N_0_	56 (38.9)	11 (22.0)	0.030^*^	29 (46.0)	6 (27.3)	0.124	0.289
N_1-2_	88 (61.1)	39 (78.0)		34 (54.0)	16 (72.7)		

PNI-, patients without pathologic perineural invasion; PNI+, patients with pathologic perineural invasion; CEA, carcinoembryonic antigen; CA19-9, carbohydrate antigen 19-9; DIS, the distance from the end of the convex edge of the tumor to the edge of the anus; CRM, circumferential resection margin; mrEMVI, MRI-based extramural vascular invasion. Data are presented as counts or means (standard deviations in parentheses). P_intra_ is the result of univariate analyses between the PNI+ and PNI- groups while P_inter_ represents whether a significant difference exists between the training and test datasets. *P < 0.05.

### Construction of Different Models

**Figure 2:**
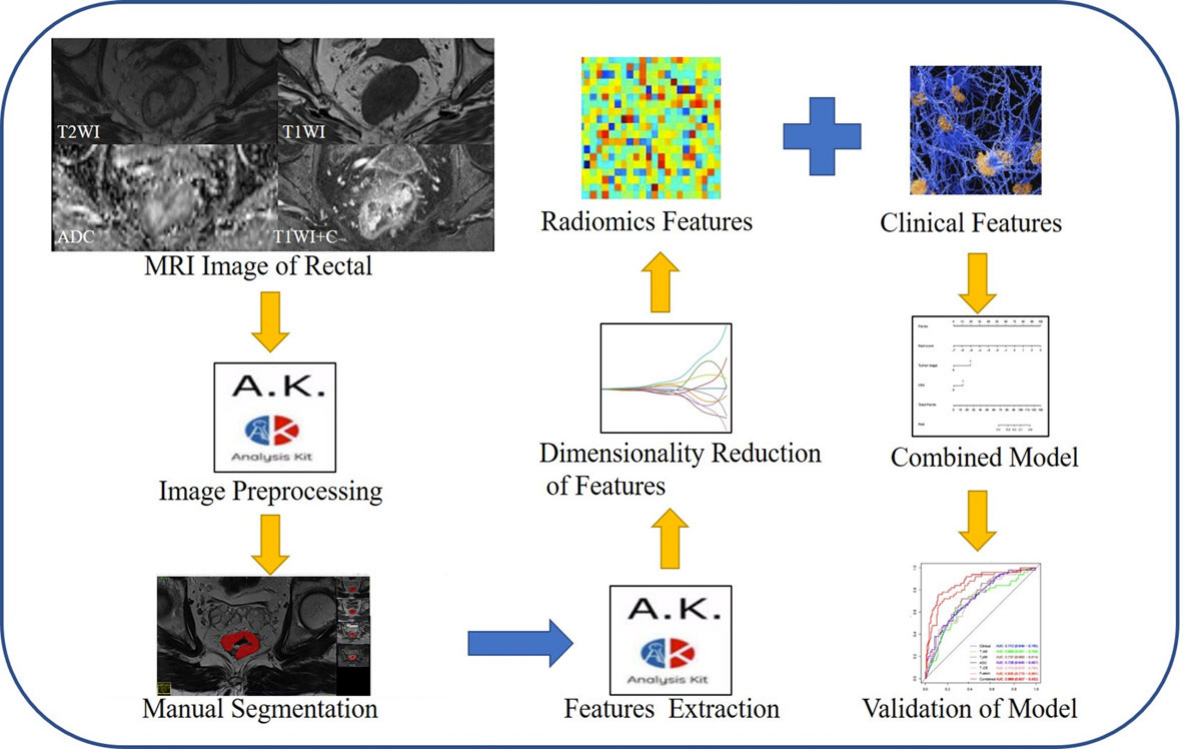
The radiomics workflow.

**Figure 2** shows the radiomics workflow. In this study, 20 features were ultimately retained from the four sequences—T_1_WI (*n* = 4), T_2_WI (*n* = 5), ADC (*n* = 5), and T_1_CE (*n* = 6)—to construct the respective single-sequence radiomics signature. Moreover, 20 features from the four sequences were used to construct the fusion radiomics signature. Details of the remaining features are described in **Supplementary Data 5**. The AUCs of T_1_WI, T_2_WI, ADC, T_1_CE, and fusion radiomics signature were 0.680, 0.737, 0.726, 0.713 and 0.835 in the training dataset, and 0.674, 0.698, 0.709, 0.666 and 0.773 in the test dataset, respectively.

Multivariate logistic regression analysis showed that CEA (odds ratio (OR) 2.749; 95% confidence interval (CI) 1.225–6.173; *P* = 0.014) and tumour stage (OR 5.956; 95% CI 1.633–21.719; *P* = 0.007) were independent predictors of PNI status (**Table 3**) and were used to construct the clinical model. The AUCs of clinical model were 0.712 and 0.690 in the training and test datasets, respectively. Moreover, CEA (*P* = 0.014), tumour stage (*P* = 0.007), and the rad-score of the fusion radiomics signature (OR 2.512; 95% CI 1.808–3.491; *P* < 0.001) were selected as independent predictors to construct the combined model. The AUCs of combined model were 0.869 and 0.864 in the training and test datasets, respectively. More information can be found in **Table 4** and **Supplementary Data 6**.

**Table 3:** Results of univariate and multivariate logistic regression analyses.

Variables	Univariate logistic regression		Multivariate logistic regression	
	OR (95%CI)	*P* value	OR (95%CI)	*P* value
Age	0.964 (0.922, 1.008)	0.106	NA	NA
Gender	1.997 (0.777, 5.135)	0.151	NA	NA
CEA	3.725 (1.468, 9.452)	0.006*	2.749 (1.225, 6.173)	0.014*
CA19-9	0.572 (0.149, 2.190)	0.415	NA	NA
DIS	1.013 (0.885, 1.159)	0.854	NA	NA
CRM status	1.623 (0.596, 4.418)	0.343	NA	NA
mrEMVI status	1.377 (0.476, 3.988)	0.555	NA	NA
Tumor stage	5.446 (1.300, 22.814)	0.020*	5.956 (1.633, 21.719)	0.007*
Lymph node	0.779 (0.260, 2.330)	0.654	NA	NA
Rad-score	2.758 (1.878, 4.051)	0.000*	2.512 (1.808, 3.491)	0.000

CEA, carcinoembryonic antigen; CA19-9, carbohydrate antigen 19-9; DIS, the distance from the end of the convex edge of the tumor to the edge of the anus; CRM, circumferential resection margin; mrEMVI, MRI-based extramural vascular invasion; OR, odds ratio; CI, confidence interval. *P < 0.05; NA, not available.

**Table 4:** Predictive performance of different models.

Items	Training dataset (n=194)				Test dataset (n=85)			
	AUC (95% CI)	Sensitivity	Specificity	Accuracy	AUC (95% CI)	Sensitivity	Specificity	Accuracy
Clinical Model	0.712 (0.640-0.785)	0.546	0.730	0.742	0.690 (0.577-0.803)	0.520	0.792	0.741
T_1_WI Signature	0.680 (0.591-0.768)	0.120	0.972	0.753	0.674 (0.539-0.808)	0.136	0.952	0.741
T_2_WI Signature	0.737 (0.660-0.814)	0.180	0.944	0.747	0.698 (0.580-0.816)	0.273	0.921	0.753
ADC Signature	0.726 (0.645-0.807)	0.240	0.958	0.773	0.709 (0.574-0.843)	0.182	0.968	0.765
T_1_CE Signature	0.713 (0.632-0.794)	0.200	0.951	0.758	0.666 (0.530-0.802)	0.318	0.921	0.765
Fusion Signature	0.835 (0.770-0.901)	0.480	0.917	0.804	0.773 (0.659-0.888)	0.364	0.889	0.753
Combined Model	0.869 (0.807-0.932)	0.620	0.924	0.845	0.864 (0.772-0.957)	0.591	0.873	0.800

T_1_CE, T_1_WI contrast-enhanced sequence; Fusion, radiomics from the four sequences; Combined, incorporating effective clinical and radiological variables and rad-score from fusion radiomics signature together; AUC, area under the curve; CI, confidence interval.

### Assessment of Different Models

The clinical model and the four single-sequence radiomics signatures performed poorly. The Delong test showed that there were no significant differences among the five models in either the training or test datasets (*P* > 0.05).

The fusion radiomics signature performed better. However, there were no significant differences between the fusion radiomics signature and the clinical model or the four single-sequence radiomics signatures in the test dataset (*P* > 0.05).

**Figure 3:**
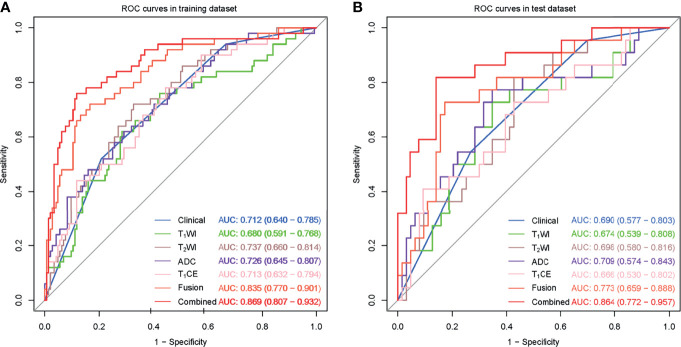
ROC curves for the perineural invasion prediction of different models in the training **(A)** and test **(B)** datasets. (Clinical, the clinical model based on effective clinical and radiological variables; T_1_CE, the T_1_CE radiomics signature based on features from T_1_WI contrast-enhanced sequence; Fusion, the fusion radiomics signature based on features from the four sequences; Combined, the combined model incorporating effective clinical and radiological variables and rad-score from fusion radiomics signature together.).

The combined model performed best. The Delong test showed that the combined model was significantly different from the clinical model and the four single-sequence radiomics signatures (*P* < 0.05). However, there were no significant differences between the combined model and the fusion radiomics signature in the training and test datasets. More information is shown in **Figure 3**.

### Validation of Combined Model

**Figure 4:**
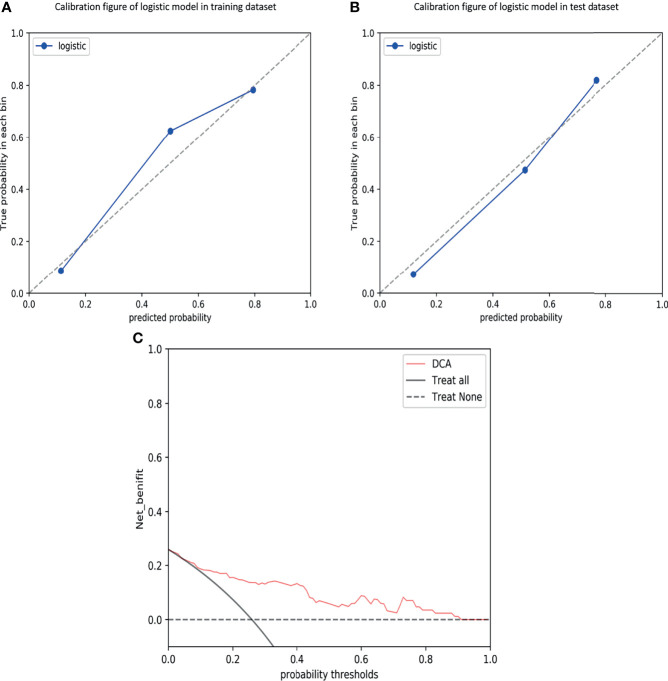
Calibration curves of the combined model in the training **(A)** and test **(B)** datasets, demonstrating good agreement between the predictions and observations. Decision curve analysis (DCA) for predicting perineural invasion status in the test dataset **(C)**, indicating good discrimination.

**Figure 5:**
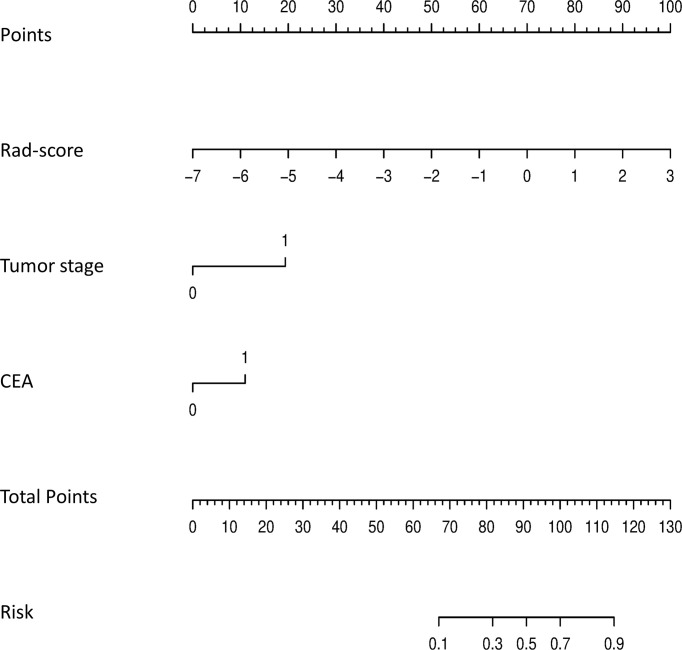
The final nomogram, including tumor stage, CEA, and rad-score, was used to predict PNI status. (PNI, perineural invasion; CEA, carcinoembryonic antigen).

**Figure 6:**
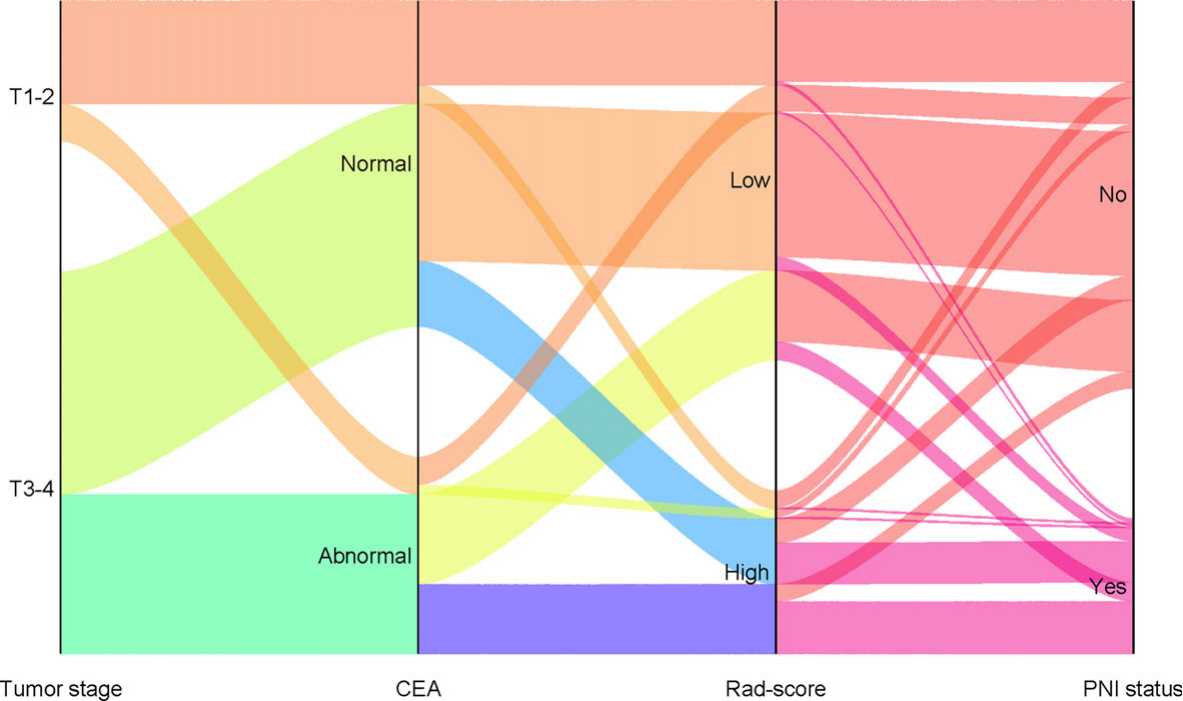
Sankey diagram showing the relationship among tumor stage, CEA, rad-score, and pathological PNI status. (PNI, perineural invasion; CEA, carcinoembryonic antigen).

The calibration curves demonstrated good agreement between the predictions and observations (**Figure 4A, B**; *P* = 0.780), indicating no deviation from normality. DCA was performed to evaluate the clinical efficiency of the combined model (**Figure 4C**). In this study, the net benefit for the combined model was higher than the measures that treat all patients and treat none patients, indicating good discrimination. The converted nomogram is shown in **Figure 5**. The relationships among tumour stage, CEA, the rad-score of the fusion radiomics signature, and pathological PNI status were disclosed and visualized as a Sankey diagram (**Figure 6**). The diagram showed that most subjects with low rad-scores had normal CEA levels and a low prevalence rate of pathological PNI, whereas subjects with high rad-scores had high tumour stages and a high prevalence rate of pathological PNI.

## Discussion

Noninvasive prognostic evaluation of RC patients has always been a hot and difficult point (22, 23). In this study, we analysed the radiomics of RC patients based on mpMRI to predict PNI status. The results showed that radiomics features extracted from multiple sequences can be better used to assess PNI status than those extracted from single sequences. In particular, the predictive nomogram constructed in combination with other clinical biomarkers had higher diagnostic performance, which further suggested that the combined model might be a powerful and noninvasive tool for predicting the PNI status of RC patients.

In rectal cancer, the recognized prognostic factors include depth of invasion, degree of differentiation, lymph node metastasis, lymphovascular invasion (LVI), and extramural vascular invasion (EMVI) (24, 25). However, previous studies have shown that the impact of PNI on prognosis is similar to the above-recognized prognostic factors (7). Therefore, PNI was introduced as an auxiliary factor in the 7th edition of the TNM classification system (26). Following this, forecasting research for PNI also began to emerge. Huang et al. (27) constructed a nomogram using clinical features to predict PNI status with AUCs of 0.704 and 0.692 in the training and test datasets, respectively, which was similar to our clinical model with AUCs of 0.712 and 0.690, respectively. However, it should be noted that the clinical features involved in model construction were different. In our study, tumour stage and CEA were the main features of clinical model construction. Previous studies have shown that the incidence of PNI increases with increasing tumour stage (19), and CEA, as an independent prognostic factor of RC, was also closely related to PNI (28). Therefore, our research further confirmed the above research conclusions.

In addition, we found that the predictive efficacies of the four single-sequence radiomics signatures were similar to or even slightly superior to that of the clinical model, although there were no significant differences. This result suggested that MRI-based radiomics features may be able to replace these clinical features in evaluating the prognosis of patients with RC, possibly benefiting from the clinical efficacy of radiomics analysis. In fact, radiomics analysis has been applied to predict pathological results, such as EMVI (29) and lymph node metastasis (30). Therefore, our research used to predict the PNI status further expanded the application scope of radiomics in RC.

There have been a few studies using radiomics to evaluate the PNI status of rectal cancer. The AUCs of the integrated model constructed by Guo et al. (31) were 0.903 and 0.889 in the training and test datasets, respectively, which were higher than the results of our combined model (AUC=0.869 and 0.864, respectively). However, what needs attention was that their integrated model combined CT-based radiomics features, and their model may not be conducive to dynamic prognostic evaluation due to radiation damage. In addition, Huang et al. (18) constructed a nomogram by combining CT radiomics with CEA, whose diagnostic efficiency was lower than that of our study. This also reflected that MRI-based radiomics was more suitable for the assessment and prediction of PNI status than CT, which may benefit from the high soft tissue resolution and no exposure to radiation (32).

Compared with the same type of research, our study also had comparative advantages. Chen et al. (33) retrospectively analysed 122 RC patients and found that the predictive model combined with T_2_WI-based radiomics, pathological N stage, and pathological LVI status may be helpful to evaluate PNI status (AUC=0.860 and 0.850, respectively), which was similar to the results of our combined model. However, it was worth noting that Chen’s study cannot be used to assess PNI status preoperatively, because postoperative pathological indicators were included in the model construction. Yang et al. (20) retrospectively analysed 140 RC patients and constructed a nomogram incorporating T_2_WI-based radiomics and MRI-reported tumour stage to predict PNI status with AUCs of 0.81 and 0.75 in the training and test datasets, respectively, which was not as good as our combined model based on multiple sequences with AUCs of 0.869 and 0.864 in the training and test datasets, respectively. Unlike the study of Yang et al., our results found that both tumour stage and CEA were the main features of nomogram construction. In addition, our study systematically evaluated and compared the predictive performance of PNI status by increasing the sample size and using multiple sequences individually or combined. Meanwhile, our results also showed that the diagnostic efficiency of the fusion radiomics signature was higher than that of the single-sequence radiomics signature. This can be explained by the multiparameter features containing more information, allowing for a more comprehensive characterization of the tumor (34–36), which provided a strong guarantee for the excellent results we obtained. Therefore, we recommend the use of mpMRI-based radiomics analysis for the prognostic assessment of RC patients, which can provide more valuable biomarker characteristics for the clinic.

Although our findings are interesting, it must be admitted that our research has some limitations. First, this was a single-centre retrospective study, which required a larger external verification team to further verify the performance. However, at least this study provided a theoretical basis for the noninvasive prediction of PNI status. Second, the sample size of our research was still small, and we will expand the sample in future studies. However, it was undeniable that DCA showed that the combined model had great clinical application potential in preoperatively predicting PNI status. Finally, our study lacked postoperative follow-up data, and this study did not explore the relationship between the model and survival outcomes. This may be another direction for future research.

In conclusion, this study provides a noninvasive method for preoperatively predicting PNI status. In particular, the comprehensive nomogram constructed by incorporating radiomics, tumour stage, and CEA can provide a convenient and practical tool for treatment strategy and help optimize individualized clinical decision-making in RC patients.

## Data Availability Statement

The original contributions presented in the study are included in the article/**Supplementary Material**. Further inquiries can be directed to the corresponding author.

## Ethics Statement

The studies involving human participants were reviewed and approved by Ethics committee of the Zhejiang Provincial People’s Hospital. Written informed consent for participation was not required for this study in accordance with the national legislation and the institutional requirements.

## Author Contributions

ZY and MY designed the study. PJ and LJ performed the data acquisition and analysis. ZY and SZ drafted and wrote the manuscript.

## Funding

This work was supported by Public welfare projects of Zhejiang Provincial Department of science and technology (LGF21H180013).

## Conflict of Interest

The authors declare that the research was conducted in the absence of any commercial or financial relationships that could be construed as a potential conflict of interest.

## Publisher’s Note

All claims expressed in this article are solely those of the authors and do not necessarily represent those of their affiliated organizations, or those of the publisher, the editors and the reviewers. Any product that may be evaluated in this article, or claim that may be made by its manufacturer, is not guaranteed or endorsed by the publisher.
