# Supplementary material for: Preoperative Prediction of Perineural Invasion Status of Rectal Cancer Based on Radiomics Nomogram of Multiparametric Magnetic Resonance Imaging
Source: Front Oncol. 2022 Apr 11;12:828904. doi: 10.3389/fonc.2022.828904 (PMC9036372; doi:10.3389/fonc.2022.828904)
Supplement: Supplementary file 1 [file DataSheet_1.docx]

**Supplementary Materials**

**Supplementary data 1. Clinical and radiological variables**

Clinical variables include age, gender, carcinoembryonic antigen (CEA), and carbohydrate antigen 19-9 (CA19-9). We considered a CEA level greater than 5 ng/mL as abnormal and a CA19-9 level greater than 37 u/mL as abnormal.

Radiological variables include MRI-based extramural vascular invasion (mrEMVI) status, circumferential resection margin (CRM) status, distance (DIS), radiological tumor (T) stage and lymph node (N) stage. Positive mrEMVI is defined as (a) the presence of tumor signal intensity within a vascular structure, (b) expanded vessels, and (c) tumoral expansion through and beyond the vessel wall, disrupting the vessel border. Positive CRM is defined as the condition when the tumor, lymph node, EMVI, or tumoral deposits are within 1 mm of the meso-rectal fascia. DIS is defined as the distance from the end of the convex edge of the tumor to the edge of the anus. Radiological T staging are divided into T_1-2_ and T_3-4_. Lymph node metastasis is defined as (a) the short-axis diameter of suspicious lymph nodes ≥9 mm; (b) short-axis diameter between 5 and 8 mm, with more than two morphologically suspicious features (including irregular border, round shape, and heterogeneous signal); (c) short-axis diameter < 5 mm, with irregular border, round shape, and heterogeneous signal; and (d) all mucinous lymph nodes which showed T_2_WI high signal (any size). These standards are widely accepted and applied in routine clinical practice[1; 2].

Two experienced radiologists (senior radiologist and junior radiologist) with 13 and 8 years of experience in rectal MRI independently assessed the quantitative and qualitative parameters. For the purposes of this study, the quantitative measurements obtained by these two radiologists were averaged for further analysis. For qualitative parameters, these two experienced radiologists carefully reviewed all the images until a consensus was reached.

**Supplementary data 2. Image preprocessing and segmentation**

Image preprocessing including resample, intensity normalization and gray-level discretization were performed with AK software (Analysis Kit, GE Healthcare). To be more specific, image preprocessing was performed by resampling the images with a resolution of 1×1×1 mm^3^ through the linear interpolation method and by discretizing and normalizing the image gray level to order 32. Extracted texture features were standardized, which removed the unit limits of the data of each feature and converted it into a dimensionless pure value. This allowed the indexes of different units or orders to be compared and weighted. We used a z-score normalization to make the image intensities fit a standard normal distribution with $\mu\text{=0}$ and $\sigma\text{=1}$, where $\mu$ is the mean value of the images, and $\sigma$ is the standard deviation. The normalized values (also called z-scores) of the image intensities (*x*) were calculated as follows:

$$z=\frac{x-\mu}{\sigma}$$

Image segmentation program is as follows. Firstly, A.K. software was used to rigorously register the images of T_2_WI, T_1_WI, ADC, and T_1_CE sequences in order to reduce the potential influence of the parameters of a scanning scheme. After that, the standardized T_2_WI images were imported into the ITK software to manually segment the entire rectal tumor layer by layer and to determine the volume of interest (VOI). Since the four sequences have been rigorously registered, tumor VOI obtained from T_2_WI can be applied directly to other sequences. All cases undertook the same VOI segmentation method.


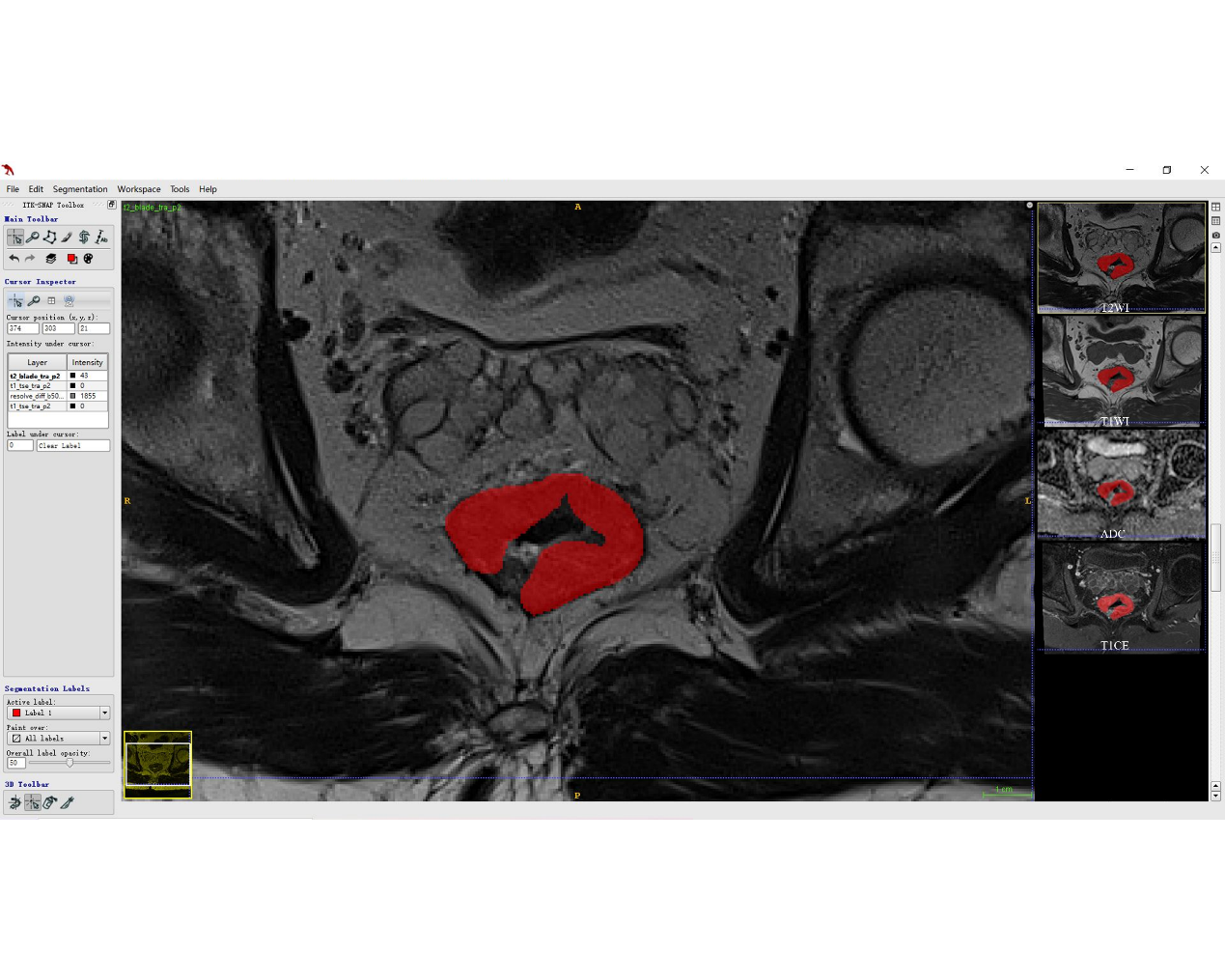


**Figure S1.** Representative manual segmentation of the entire rectal tumor in the T_2_WI, T_1_WI, ADC, and T_1_CE using ITK software.

**Supplementary data 3. All radiomics features**


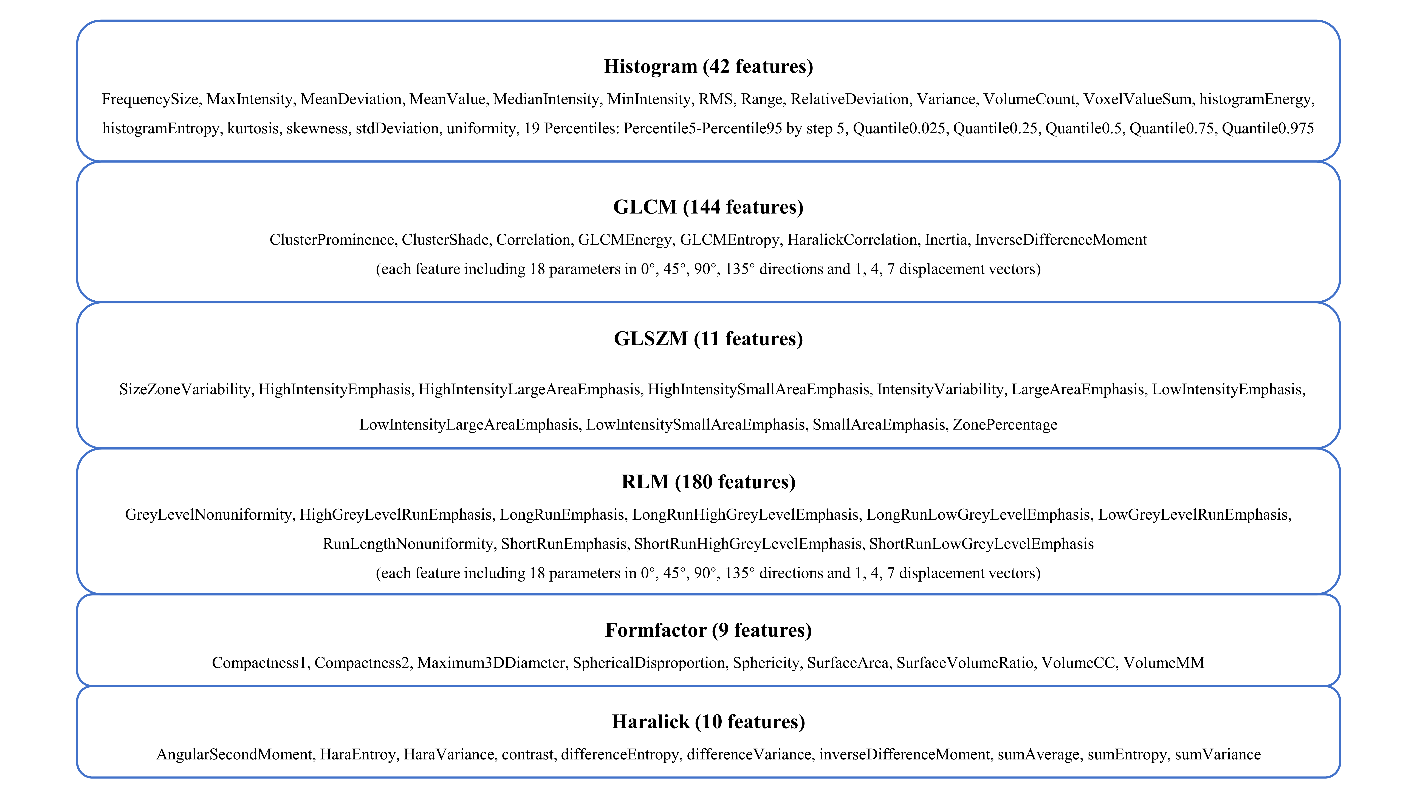


**Figure S2.** Detailed information of all radiomics features

**Supplementary data 4. Radiomics features selection**

A total of 1584 radiomics features were extracted for each patient from the four sequences, and 1097 were retained by detection of robustness and reproducibility using Spearman’s rank correlation test. Then, analysis of variance was used to perform preliminary dimension reduction on these features. The variance value is the average of the square of the difference between the value of each variable and the mean. It is the most important method for measuring the dispersion of numeric data. The larger the variance, the greater the fluctuation of the data, and vice versa. So, it is necessary to preferentially eliminate features with a variance of 0 or less. In this study, the variance of each feature was calculated, and then the features greater than the threshold 1 were retained. Following analysis of variance, 677 features were selected. And after that, least absolute shrinkage and selection operator (LASSO) was used for ultimate dimension reduction. LASSO is a powerful algorithm for regression analysis with high dimensional predictors. The LASSO algorithm shrinks some coefficients and reduces others to exactly 0 via an absolute constraint. Thus, LASSO is an outstanding method for feature selection as it retains good features using both a subset selection and ridge regression. Finally, 20 features were ultimately retained from the four sequences—T_1_WI (*n* = 4), T_2_WI (*n* = 5), ADC (*n* = 5), and T_1_CE (*n* = 6).

**
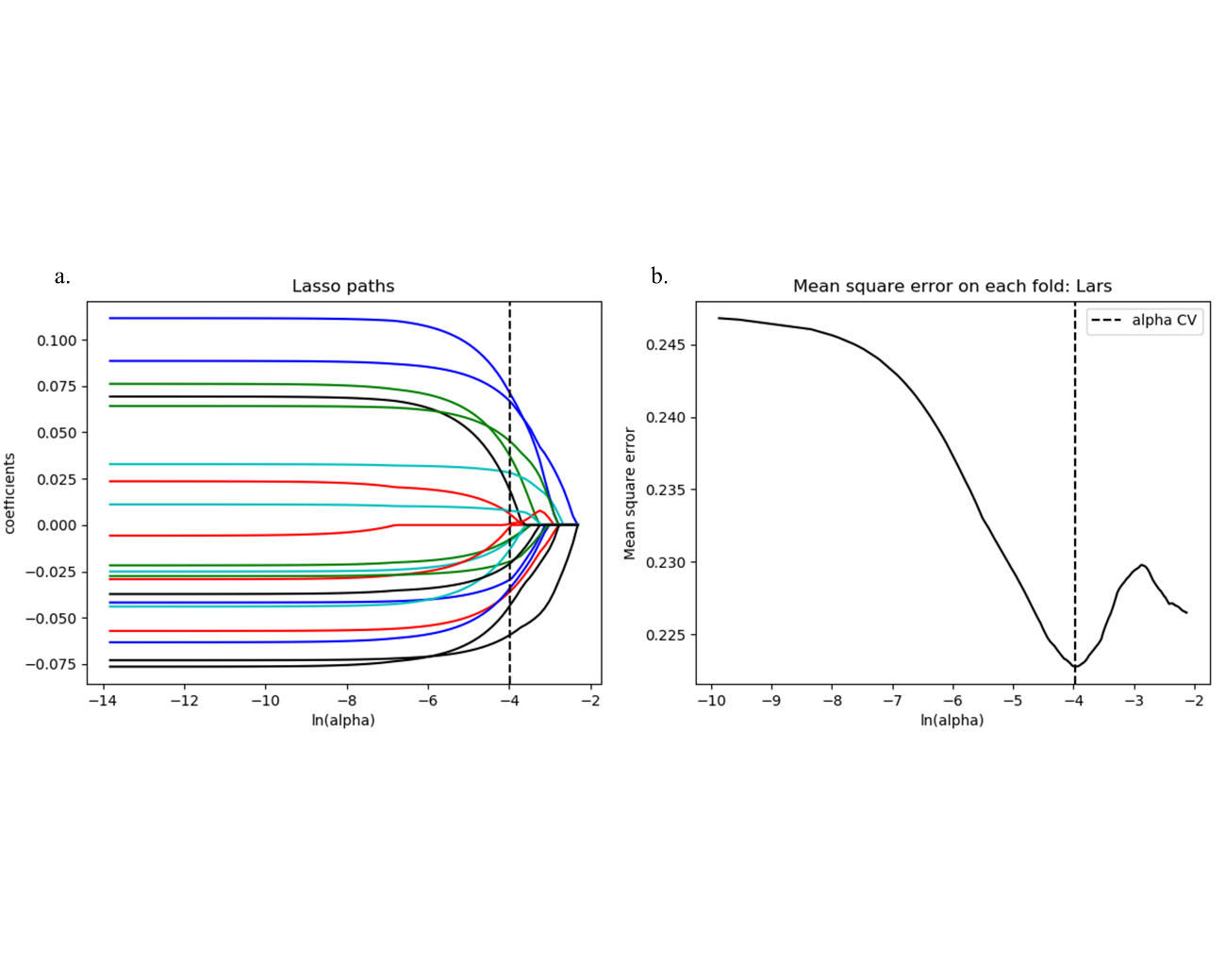
**

**Figure S3.** Radiomics feature selection. (a) LASSO coefficient profiles of radiomics features. (b) Mean square error on each fold for radiomics feature selection with LASSO.

**Supplementary data 5.** **Remaining radiomics features**

**Table S1.** The detailed information of remaining radiomics features

| **Sequence** | **Coefficient** | **Feature** | **Category** |
| --- | --- | --- | --- |
| T_1_WI (n=4) | -0.506 | ClusterShade_angle90_offset7 | GLCM |
|  | 0.116 | GLCMEnergy_angle90_offset7 | GLCM |
|  | -0.109 | InverseDifferenceMoment_angle90_offset7 | Haralick |
|  | 0.246 | ShortRunHighGreyLevelEmphasis_AllDirection_offset1_SD | RLM |
| T_2_WI (n=5) | 0.488 | VoxelValueSum | Histogram |
|  | -0.402 | ClusterProminence_angle90_offset1 | GLCM |
|  | 0.516 | LongRunEmphasis_angle45_offset1 | RLM |
|  | -0.330 | LongRunLowGreyLevelEmphasis_angle45_offset4 | RLM |
|  | -0.514 | SurfaceVolumeRatio | Formfactor |
| ADC (n=5) | 0.719 | Variance | Histogram |
|  | -0.104 | GLCMEnergy_angle0_offset1 | GLCM |
|  | 0.375 | ShortRunHighGreyLevelEmphasis_AllDirection_offset7_SD | RLM |
|  | 0.086 | ShortRunLowGreyLevelEmphasis_angle135_offset1 | RLM |
|  | -0.226 | LowIntensitySmallAreaEmphasis | GLSZM |
| T_1_CE (n=6) | -0.537 | VoxelValueSum | Histogram |
|  | -0.347 | ClusterProminence_angle0_offset7 | GLCM |
|  | -0.251 | ClusterProminence_angle45_offset4 | GLCM |
|  | -0.203 | HaralickCorrelation_angle45_offset7 | GLCM |
|  | -0.322 | InverseDifferenceMoment_angle90_offset1 | Haralick |
|  | 0.450 | RunLengthNonuniformity_AllDirection_offset7_SD | RLM |

Note. Rad-score of the fusion radiomics signature can be calculated by intercept and their respective coefficients. intercept = -1.515.

**Supplementary data 6. Calculation formula for the combined model**

*model-score of combined model* = -1.919+0.921×rad-score+1.784×tumor stage+1.011×CEA


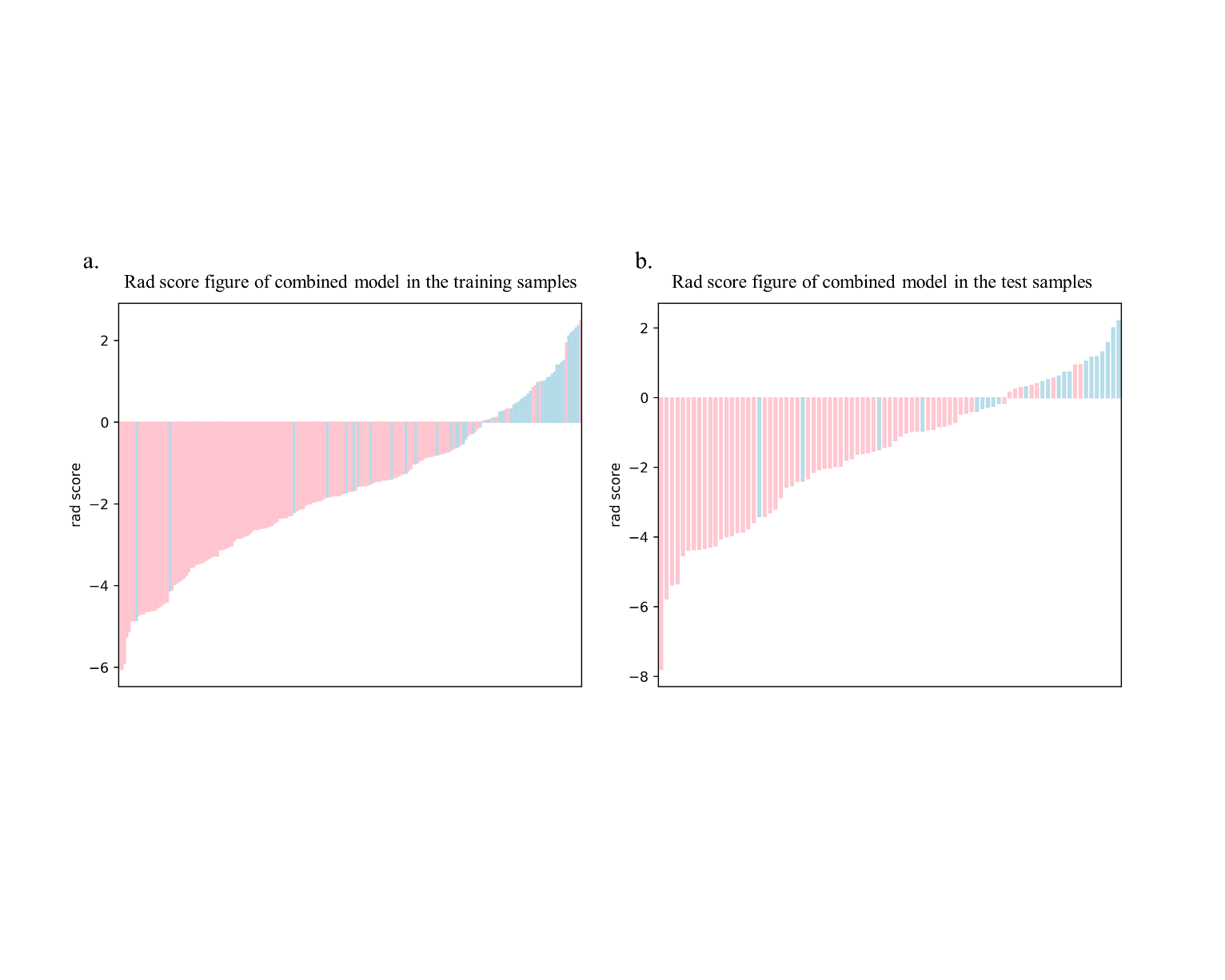


**Figure S4**. Rad score figures of combined model in the training (a) and test (b) datasets. Red represents PNI- set and blue represents PNI+ set. A score greater than 0 indicates PNI+, and a score less than 0 indicates PNI-.

**Reference**

1 Horvat N, Carlos Tavares Rocha C, Clemente Oliveira B, Petkovska I, Gollub MJ (2019) MRI of Rectal Cancer: Tumor Staging, Imaging Techniques, and Management. Radiographics 39:367-387

2 Nougaret S, Jhaveri K, Kassam Z, Lall C, Kim DH (2019) Rectal cancer MR staging: pearls and pitfalls at baseline examination. Abdom Radiol (NY) 44:3536-3548
